# Silent threat after surgery: subclavian and axillary vein thrombosis following clavicle fixation leading to thoracic outlet syndrome

**DOI:** 10.1016/j.radcr.2025.08.086

**Published:** 2025-09-19

**Authors:** Morteza Gholipour, Arian Hajiahmadi, Mohsen Salimi, Mahdi Mohammaditabar, Fatemeh Abbasi

**Affiliations:** a Clinical Research Development Unit of Akhtar Hospital, Shahid Beheshti University of Medical Science, Tehran, Iran; b Student Research Committee, Faculty of Medicine, Tabriz University of Medical Sciences, Tabriz, Iran; c School of Medicine, Shiraz University of Medical Sciences, Shiraz, Iran; d Student Research Committee, Faculty of Medicine, Mazandaran University of Medical Sciences, Mazandaran, Iran

**Keywords:** Clavicle fracture, Venous thrombosis, Thoracic outlet syndrome, Apixaban

## Abstract

Thoracic outlet syndrome (TOS) is a rare but significant complication that may occur following clavicular fractures, especially after surgical fixation. We present the case of a 32-year-old woman who developed partial thrombosis of the subclavian and axillary veins as a delayed complication after open reduction and internal fixation (ORIF) of a clavicle fracture with an anatomical plate. Approximately 1 month after surgery, the patient began experiencing pain in the axillary region and mid-arm, symptoms that raised suspicion for TOS. Doppler ultrasonography confirmed partial thrombosis of the distal subclavian vein and acute thrombosis extending from the axillary vein to the subclavian junction. Anticoagulation therapy with apixaban (2.5 mg every 12 hours) was initiated, resulting in significant symptomatic improvement within 1 month, although mild axillary discomfort persisted. Long-term follow-up over 11 months revealed complete resolution of symptoms, and repeat Doppler imaging showed full restoration of venous flow with no residual thrombosis. This case emphasizes the need for clinical vigilance regarding vascular complications after clavicular surgery and demonstrates the therapeutic potential of apixaban in managing venous TOS. Early recognition and timely anticoagulation may lead to favorable outcomes in similar postoperative cases.

## Introduction

Clavicular injuries are notably prevalent, comprising approximately 10% of all fracture cases [1]. Thrombotic events associated with clavicle fractures are uncommon, with the majority of occurrences linked to penetrating trauma or high-energy mechanisms [[2], [3], [4]]. Venous thromboembolisms (VTEs) involving the upper extremities account for only 1–4% of all VTE incidents [5]. Furthermore, there is little evidence to support an association between VTE and upper limb immobilization, such as that used in treating clavicle fractures with slings [5]. As a result, there are no formal guidelines for VTE prevention in the context of upper limb immobilization [5]. Prophylactic interventions for VTE are warranted only in cases of upper extremity fractures accompanied by specific risk factors, such as hypercoagulability or prolonged bed rest [5].

Paget-Schroeter syndrome (PSS) is a thrombosis caused by the effort of the axillary and subclavian veins with compression of the subclavian vein at the thoracic outlet. This venous variant is thoracic outlet syndrome (TOS), a syndrome of symptoms associated with compression of the subclavian vein, subclavian artery, or brachial plexus as it passes through the thoracic outlet [[6], [7], [8], [9], [10]].

## Case presentation

**Fig. 1:**
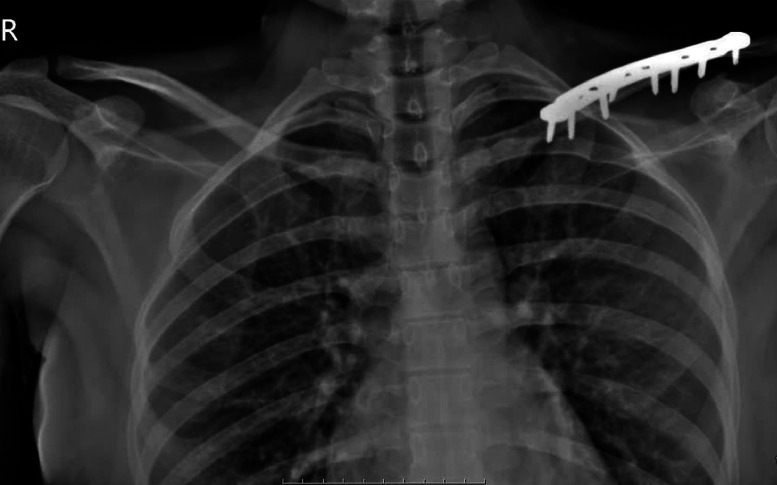
Postoperative X-ray of the clavicle after ORIF with plates and screws.

A 32-year-old woman presented to the emergency department following a motor vehicle accident, which resulted in a fracture of the left clavicle. The patient was stable, with vital signs as follows: blood pressure 125/80 mmHg, heart rate 83 beats/min, respiratory rate 18 breaths/min, temperature 36.7°C, and oxygen saturation 96% on room air. All laboratory data for the patient were within normal limits. The patient had no past medical history, no underlying medical conditions, no prior surgeries, no significant family history, and was not taking any medications. An X-ray of the shoulder was performed, which confirmed the presence of a displaced clavicle fracture, with no accompanying injury or additional fractures at other sites. After initial imaging and discussion of treatment options, surgical intervention was planned 2 days later. The patient underwent open reduction and internal fixation (ORIF) using an anatomical plate and screws to stabilize the fracture (Fig. 1). The patient underwent surgery without complications and was discharged 2 days later.

**Fig. 2:**
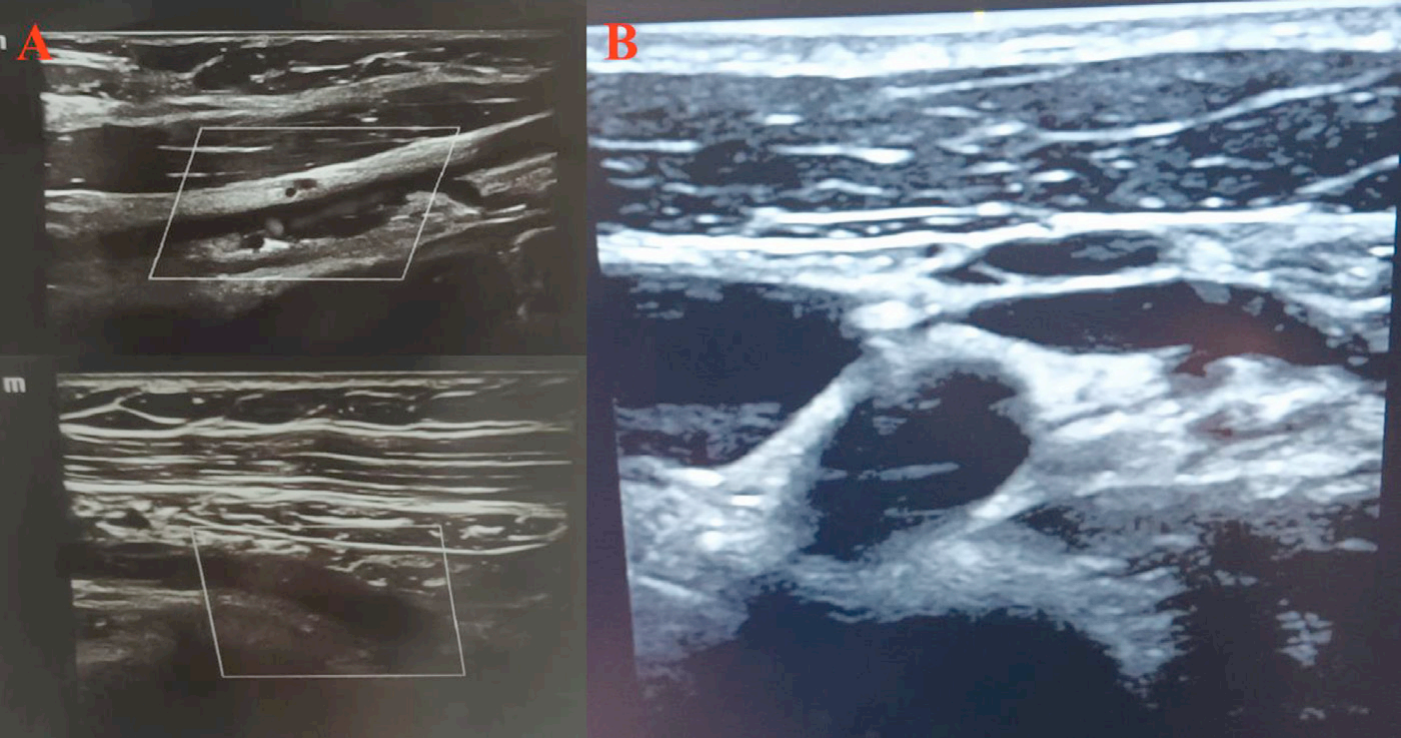
Color Doppler ultrasound (A) revealing subacute thrombosis in the distal subclavian vein and the upper third of the axillary vein, extending to the junction with the subclavian vein and (B) After maintaining apixaban treatment for eleven months.

Approximately 1 month after surgery, she reported pain in the axillary region and the midportion of her arm, with a sensation of heaviness in the arm. On clinical examination, her symptoms initially suggested thoracic outlet syndrome (TOS); however, there were no findings of coldness, pallor, color changes in the hand, weak or absent pulses, cyanosis, or swelling. Further evaluation with Color Doppler ultrasound demonstrated subacute partial thrombosis of the distal subclavian vein and the upper third of the axillary vein, extending to the subclavian–axillary junction (Fig. 2). The patient was started on apixaban, 2.5 mg twice daily. After 1 month of anticoagulation therapy, her symptoms showed marked improvement, with significant relief of pain and restoration of functional activity; however, she continued to report mild discomfort in the axillary region.

With continued anticoagulation therapy over the subsequent ten months, the patient experienced complete resolution of symptoms. A follow-up Color Doppler ultrasound performed at the end of this period demonstrated full recanalization of the previously affected veins, with no evidence of residual thrombosis (Fig. 2). At the 1-year follow-up visit, the patient exhibited complete clinical recovery, and imaging findings corroborated the absence of vascular abnormalities.

## Discussion

This case report describes a patient who developed subacute partial thrombosis near the clavicle and axillary region following surgical fixation of a clavicle fracture through ORIF.

About 1 month after surgery, the patient presented with pain in the axillary region and mid-arm. Initially, the symptoms were suggestive of thoracic outlet syndrome due to the proximity of the fracture to that area; however, Color Doppler ultrasound revealed subacute thrombosis, confirming VTE instead. This diagnostic shift emphasizes the importance of maintaining a broad differential diagnosis when evaluating postoperative pain, rather than focusing solely on the usual causes.

Clavicle fractures can occasionally result in neurovascular complications, such as thoracic outlet syndrome [11,12]. However, this case highlights that VTE should also be considered, even though it is rare. It is imperative to suspect when pain persists or presents atypically. Color Doppler ultrasound plays a critical role in this context, serving as a noninvasive and effective tool for detecting thrombosis.

The patient was treated with apixaban, an anticoagulant, at a dose of 2.5 mg twice daily to manage the clot. Anticoagulation remains the cornerstone of therapy for upper extremity deep vein thrombosis (DVT) [13]. After 1 month of treatment, the patient’s symptoms improved, and ultrasound findings showed partial resolution, although mild axillary pain persisted. Continued apixaban therapy for an additional ten months resulted in complete symptom resolution and normalization of ultrasound findings at the 1-year follow-up.

The treatment in this case is consistent with guidelines recommending direct oral anticoagulants (DOACs) for the management of VTE [13]. These medications are preferred because they are effective, safe, and easy to administer. In this case, the patient received a DOAC for 12 months, which is longer than the typical 3 to 6 months recommended for upper extremity thrombosis [13]. This extended treatment duration was likely due to the persistence of mild symptoms, prompting clinicians to take additional precautions to ensure complete clot resolution, even though guidelines vary regarding the optimal treatment length for thrombosis in this region. The occurrence of a blood clot following clavicle fracture repair is rare [14].

Adla et al. [15] reported another case in which a patient developed subclavian vein thrombosis following a clavicle fracture that was managed nonsurgically. This suggests that the fracture itself may cause vascular injury and trigger thrombogenesis, rather than surgery being the sole contributing factor. The development of thrombosis may be attributed to several factors, including vascular injury from the initial trauma, venous stasis due to restricted upper limb mobility, or the surgical intervention itself, specifically ORIF [16].

This paper also emphasizes the need for continued vigilance following the initial clinical assessment, particularly the necessity of considering VTE in the differential diagnosis after clavicle fracture repair. Rapid diagnosis using Color Doppler ultrasound, combined with effective anticoagulant therapy with apixaban, resulted in a highly favorable outcome, with the patient fully recovering within 1 year. This case demonstrates that early recognition and appropriate treatment of such uncommon complications can significantly improve outcomes.

## Conclusion

This case underscores the potential for vascular complications, such as partial thrombosis of the subclavian and axillary veins, following clavicular fractures and surgical intervention. The successful management of this patient’s condition with apixaban illustrates its effectiveness in treating postsurgical venous thrombosis associated with thoracic outlet syndrome. Ongoing monitoring and individualized treatment are crucial to ensuring favorable outcomes. As awareness of these complications increases, further research is warranted to refine management protocols and improve patient care in similar cases.

## Patient consent

Written informed consent was obtained from the patient for publication and any accompanying images. A copy of the written consent is available for review by the Editor-in-Chief of this journal on request
